# Supplementary material for: Aerosol forcing regulating recent decadal change of summer water vapor budget over the Tibetan Plateau
Source: Nat Commun. 2024 Mar 12;15:2233. doi: 10.1038/s41467-024-46635-8 (PMC10933345; doi:10.1038/s41467-024-46635-8)
Supplement: Supplementary file 1 — Supplementary Information [file 41467_2024_46635_MOESM1_ESM.pdf]

Supplementary Information

for

Aerosol forcing regulating recent decadal change of summer water vapor budget over the Tibetan Plateau

Supplementary Table 1 Summary of eleven CMIP6 models used in this study.

Model	Country	Number of grid (Lon × Lat)
ACCESS-CM2	Australia	192×144
ACCESS-ESM1-5	Australia	192×144
BCC-CSM2-MR	China	320×160
CanESM5	Canada	128×64
CNRM-CM6-1	France	256×128
E3SM-2-0	USA	360×180
HadGEM3-GC31-LL	UK	192×144
IPSL-CM6A-LR	France	144×143
MIROC6	Japan	256×128
MRI-ESM2-0	Japan	320×160
NorESM2-LM	Norway	144×96

Supplementary Table 2 Simulated linear trends (unit: 10^6 kg s^{-1} decade^{-1}) of water vapor budget in eastern boundary of TP during 1979-2014 from eleven CMIP6 models.

Model	Trend
ACCESS-CM2	1.1
ACCESS-ESM1-5	2.5
BCC-CSM2-MR	0.8
CanESM5	-0.3
CNRM-CM6-1	0.5
E3SM-2-0	1.4
HadGEM3-GC31-LL	2.0
IPSL-CM6A-LR	0.5
MIROC6	0.4
MRI-ESM2-0	2.6
NorESM2-LM	-2.3

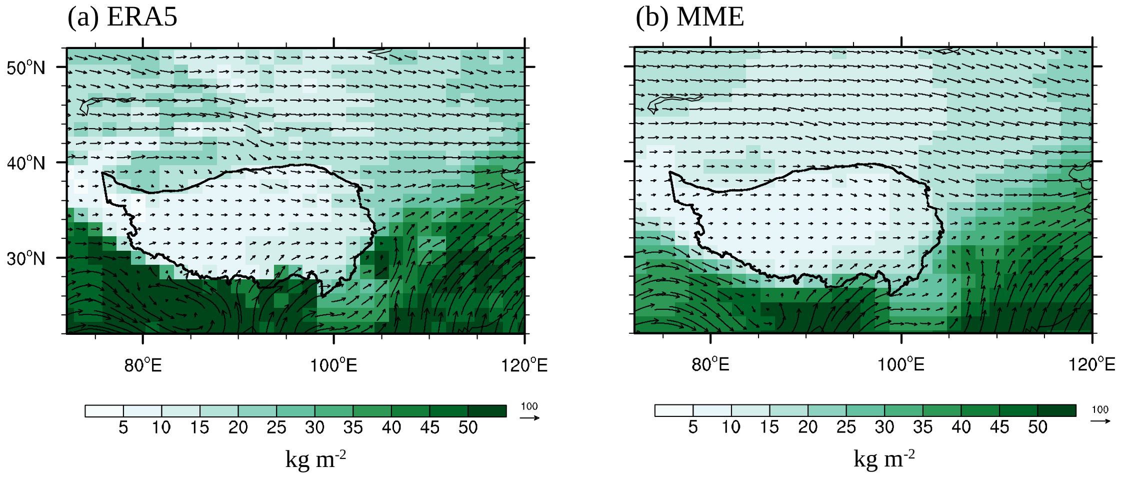

Supplementary Fig. 1 | Vertically integrated precipitable water vapor and water vapor flux. Spatial pattern of climatological vertically integrated precipitable water vapor (shade, unit: kg m^{-2}) and water vapor flux (vector, unit: kg m^{-1} s^{-1}) over the Tibetan Plateau during 1979-2014 from observation **(a)** and all forcing simulation **(b)**. MME represents multi-model ensemble mean.

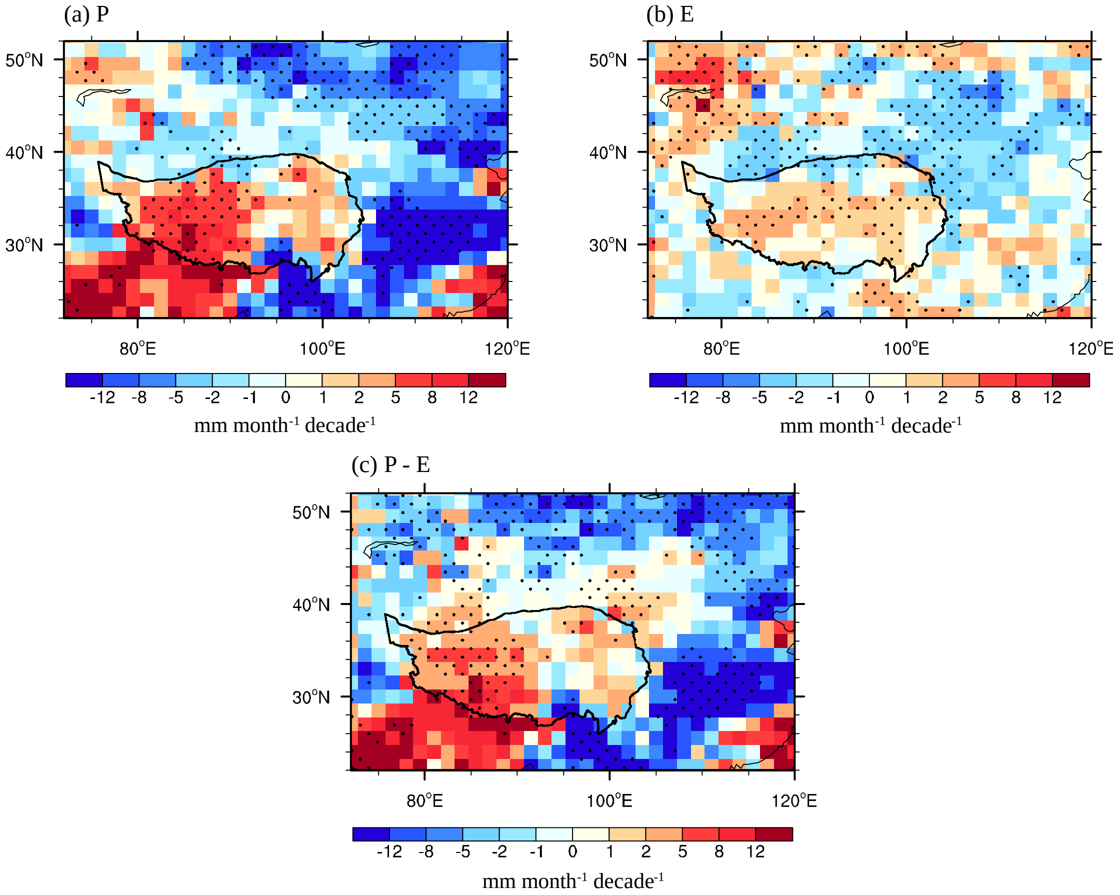

Supplementary Fig. 2 | Linear trends of summer mean precipitation and evaporation. Observed linear trends of summer mean precipitation (P, **a**), evaporation (E, **b**) and its difference ($P-E$, **c**) during 1979-2014. The black points represent statistical significance at 90% confidence level.

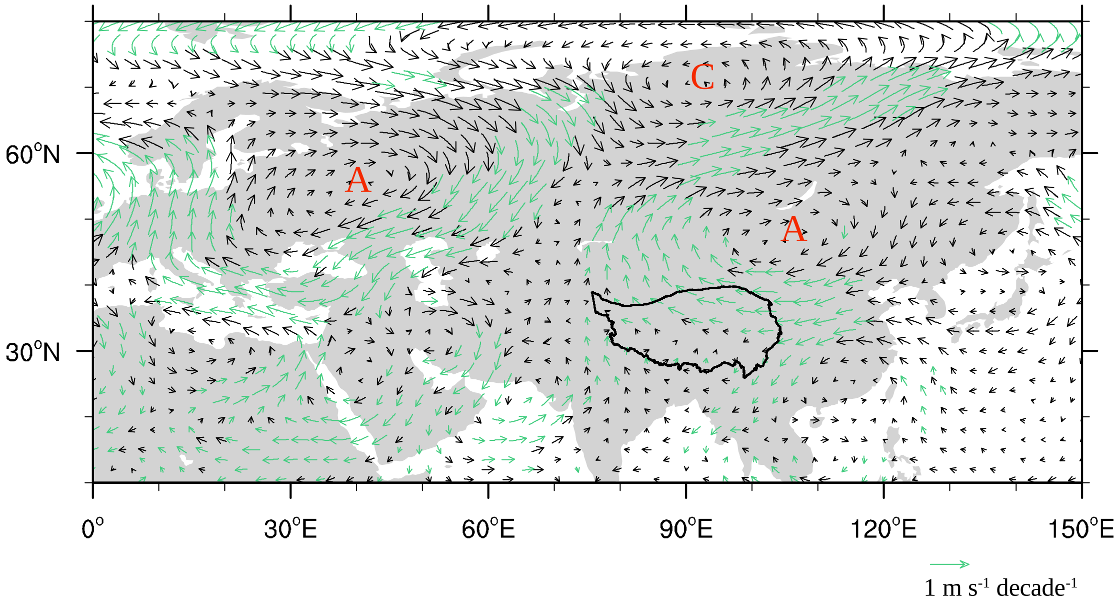

Supplementary Fig. 3 | Linear trends of summer mean wind vector. Observed linear trends of summer mean wind vector (units: m s^{-1} $decade^{-1}$) at 500 hPa during 1979-2014. The green vectors represent statistical significance at 90% confidence level.

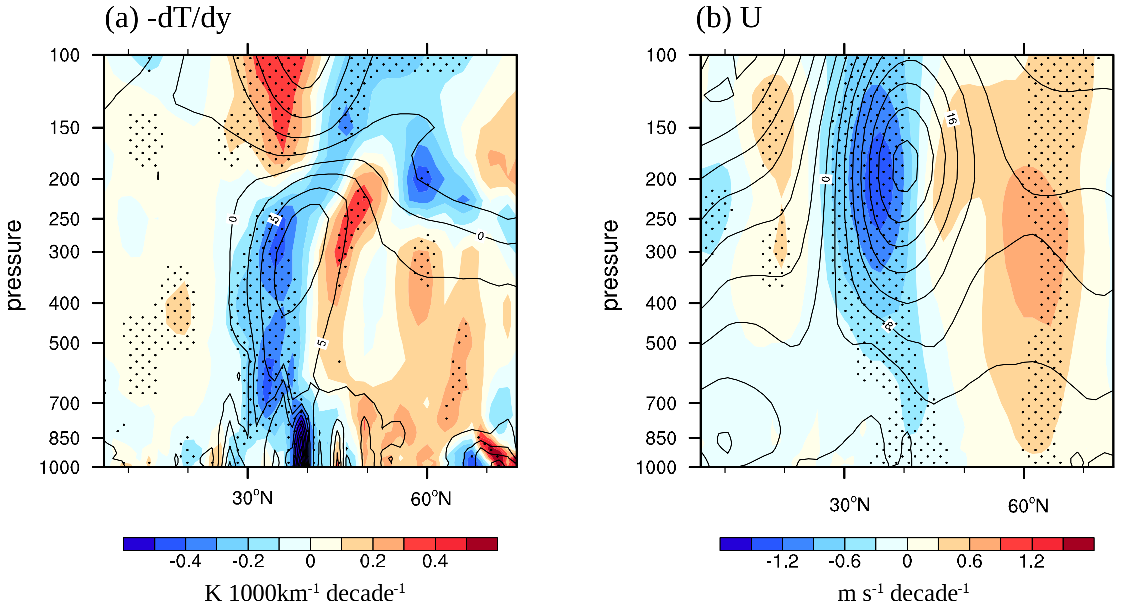

Supplementary Fig. 4 | Meridional temperature gradient and zonal winds. Observed climatology (contour) and linear trends (shade) of summer mean meridional temperature gradient (-dT/dy, unit: K $1000km^{-1}$ $decade^{-1}$) **(a)** and zonal winds (unit: m s^{-1} $decade^{-1}$) **(b)** zonally averaged between 90°E and 120°E during 1979-2014. The black points indicate statistical significance at 90% confidence level.

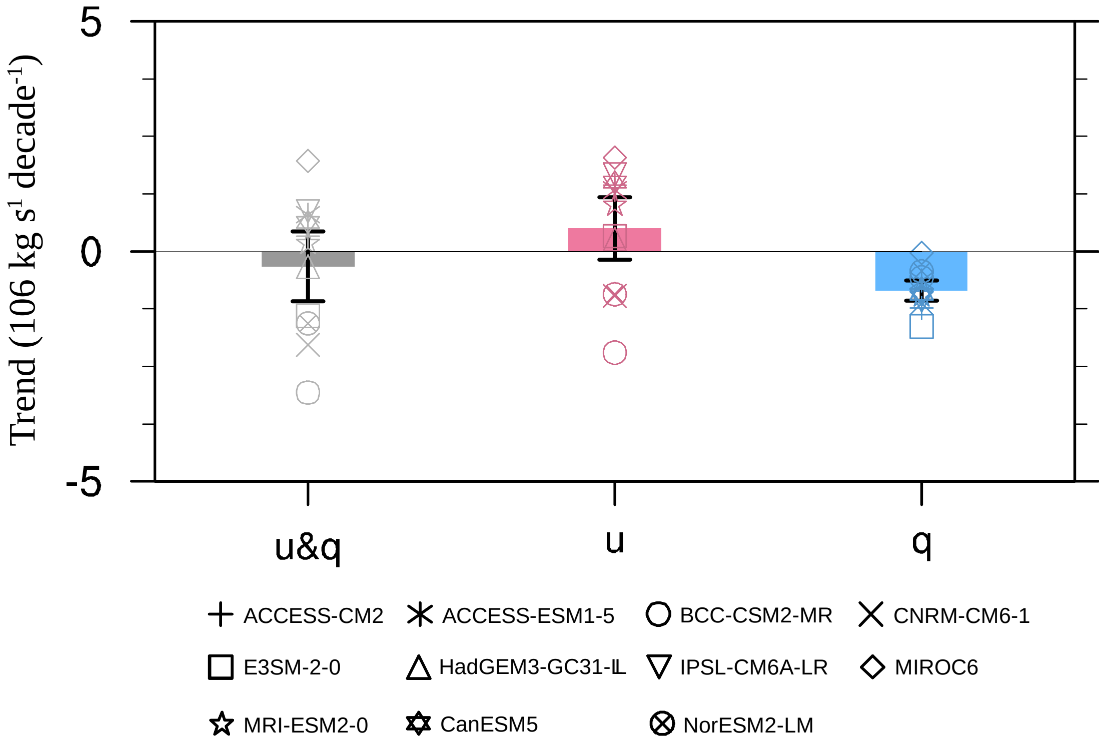

Supplementary Fig.5 | Linear trends of water vapor flux. Same as Figure 3 except for greenhouse gas forcing-only simulations.

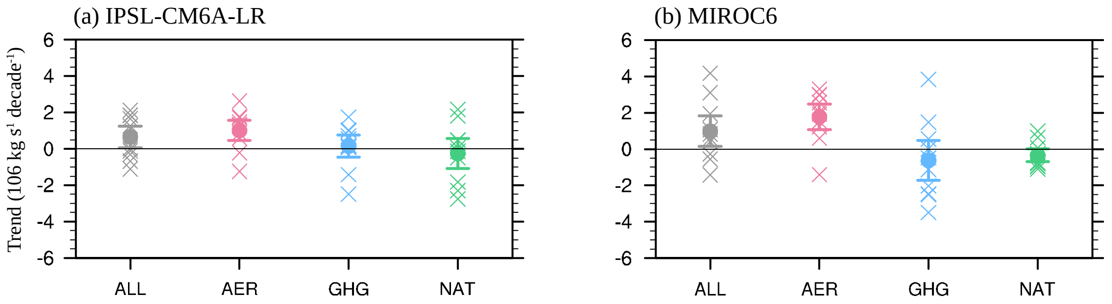

Supplementary Fig. 6 | Attribution of changes in summer water vapor flux. Attribution of changes in summer water vapor flux in the eastern boundary of the Tibetan Plateau from ten ensemble members from IPSL-CM6A-LR and MIROC6. The gray, red, blue and green markers represent simulated linear trends (unit: 10^6 kg s^{-1} decade^{-1}) of water vapor flux in eastern boundary of the Tibetan Plateau during 1979-2014 from all (ALL), aerosol-only (AER), greenhouse gas-only (GHG) and natural-only (NAT) forcings simulations in ten ensemble members, respectively. The points and error bars represent ensemble means and one standard deviations of ten ensemble members for each model.

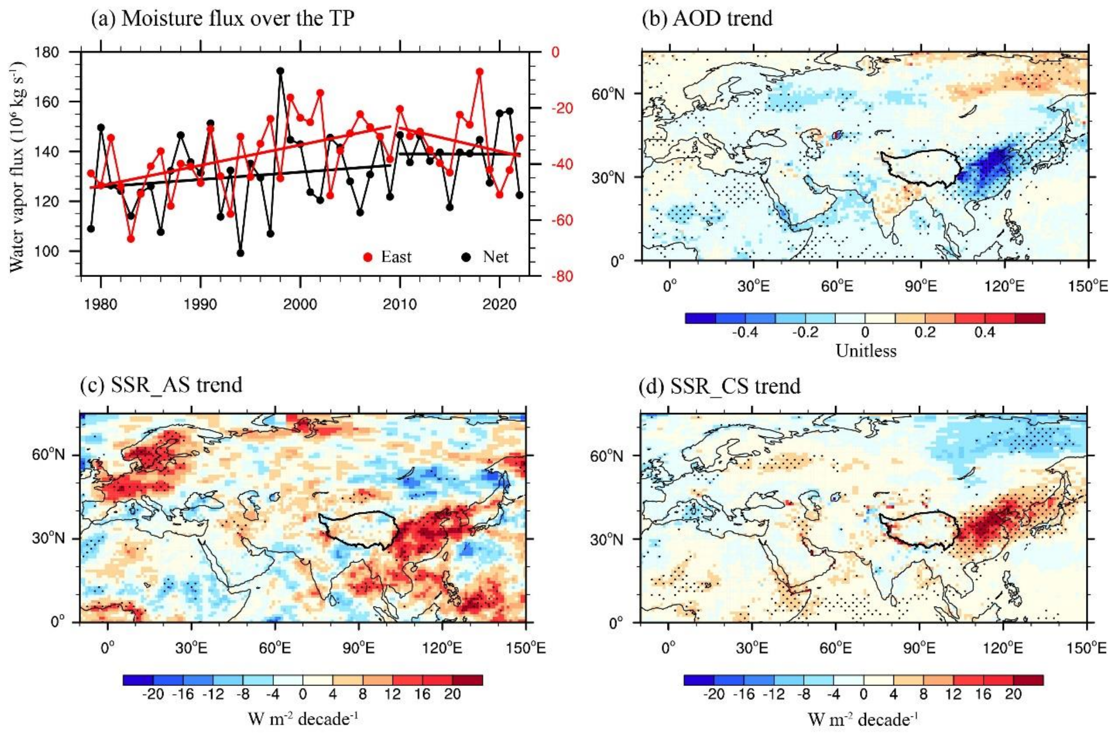

Supplementary Fig. 7 | Water vapor flux, aerosol optical thickness and surface solar radiation. (a) Observed time series (point) and linear fitting (line) of summer mean water vapor flux (unit: 10^6 kg s^{-1}) in the Tibetan Plateau during 1979-2014. **(b-d)** Linear trends (shade) of summer mean aerosol optical thickness (AOD, unit: unitless) and surface solar radiation for all-sky and clear-sky conditions (SSR_AS and SSR_CS, unit: W m^{-2} $decade^{-1}$) during 2010-2022. The black points in panels (b-d) indicate statistical significance at 90% confidence level.

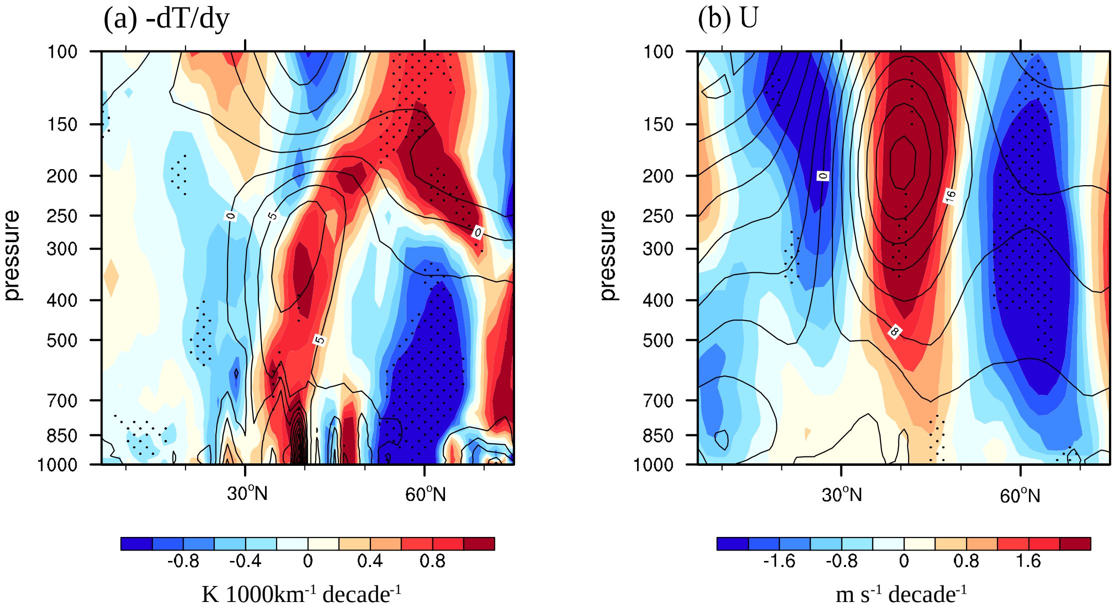

Supplementary Fig. 8 | Meridional temperature gradient and zonal winds. Observed climatology (contour) and linear trends (shade) of summer mean meridional temperature gradient (-dT/dy, unit: K $1000km^{-1}$ $decade^{-1}$) **(a)** and zonal winds (unit: m s^{-1} $decade^{-1}$) **(b)** zonally averaged between 90°E and 120°E during 2010-2022. The black points indicate statistical significance at 90% confidence level.

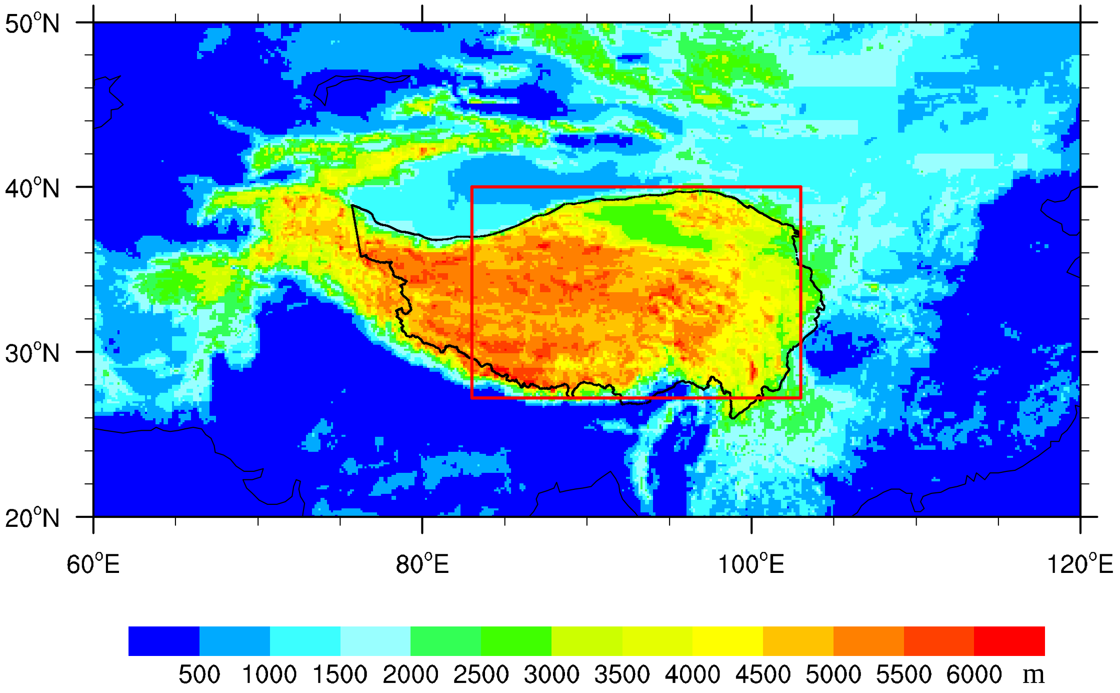

Supplementary Fig. 9 | Study area. Four boundaries (Red lines) used for calculation of water vapor budget over the Tibetan Plateau. The shadings represent the elevation.
